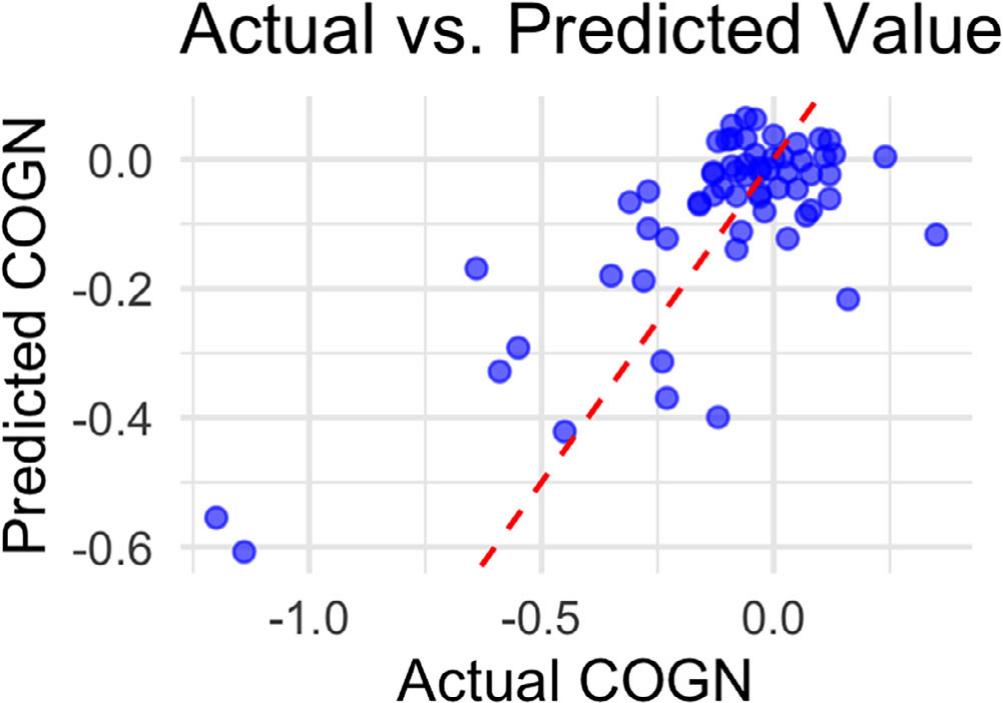# Functional and Emotional Predictors of Cognitive Decline: Insights from the ADNI Dataset

**DOI:** 10.1002/alz70857_100984

**Published:** 2025-12-24

**Authors:** Nicola Sambuco, Giorgia Francesca Scaramuzzi, Daphne Gasparre, Ester Cornacchia, Aurora Bonvino, Linda A. Antonucci, Giulio Pergola, Paolo Taurisano

**Affiliations:** ^1^ University of Bari Aldo Moro, Bari, Bari, Italy; ^2^ Department of Translational Biomedicine and Neuroscience “DiBraiN”, University of Bari Aldo Moro, Bari, Italy

## Abstract

**Background:**

Cognitive decline is a hallmark of aging and neurodegenerative diseases, including Alzheimer's disease, and is considered a cornerstone of early identification efforts. Previous studies have reported that cognitive decline is associated with structural changes in the brain, functional impairment (FAQ), and mood alterations (GDS). However, understanding the combined predictive power of structural changes in the brain, functional impairment (FAQ), and mood alterations (GDS) is critical for identifying their roles as determinants of cognitive decline, thereby improving early detection and targeted interventions.

**Method:**

We analyzed data from the Alzheimer's Disease Neuroimaging Initiative (ADNI) to identify predictors of cognitive decline using the Montreal Cognitive Assessment (MoCA), a tool that evaluates multiple cognitive domains, in participants with a score ≥4 (*n* = 312). The dependent variable, cognitive change, was quantified as the beta coefficient obtained from the regression analysis of MoCA scores over time (4+ time points). LASSO regression evaluated the baseline relationships between hippocampal volume (HIPPO), functional abilities (FAQ), and depressive symptoms (GDS), with 5‐fold cross‐validation to minimize overfitting. The model was trained on 80% of the data and evaluated on 20% using Mean Squared Error (MSE), Mean Absolute Error (MAE), and *R* ^2^.

**Result:**

In predicting cognitive trajectories (COGN), the LASSO regression model achieved R2=0.51R^2 = 0.51R2=0.51 on the test set, explaining 51% of the variance in cognitive decline. FAQ accounted for the largest proportion of the explained variance (86.2%), followed by HIPPO (7.7%) and GDS (6.0%). These findings highlight the dominant role of functional abilities (FAQ) in predicting cognitive decline, with depressive symptoms (GDS) and hippocampal volume (HIPPO) contributing minimally.

**Conclusion:**

The current findings demonstrate that baseline functional and emotional measures can predict future cognitive decline, with functional difficulties (FAQ) contributing the most, followed by depressive symptoms (GDS), and hippocampal volume (HIPPO). These results suggest that functional and emotional measures vary alongside cognitive decline, with neuroimaging offering complementary insights when combined with behavioral evaluations for early detection.